# Rethinking the evidence for early horse domestication at Botai

**DOI:** 10.1038/s41598-021-86832-9

**Published:** 2021-04-02

**Authors:** William Timothy Treal Taylor, Christina Isabelle Barrón-Ortiz

**Affiliations:** 1 University of Colorado-Boulder Museum of Natural History, 218 UCB, Boulder, CO 80309 USA; 2 Quaternary Palaeontology Program, Royal Alberta Museum, 9810 103a Ave NW, Edmonton, AB T5J 0G2 Canada

**Keywords:** Archaeology, Palaeontology

## Abstract

Despite its transformative impact on human history, the early domestication of the horse (*Equus caballus*) remains exceedingly difficult to trace in the archaeological record. In recent years, a scientific consensus emerged linking the Botai culture of northern Kazakhstan with the first domestication of horses, based on compelling but largely indirect archaeological evidence. A cornerstone of the archaeological case for domestication at Botai is damage to the dentition commonly linked with the use of bridle mouthpieces, or “bit wear.” Recent archaeogenetic analyses reveal, however, that horse remains from Botai are not modern domesticates but instead the Przewalski’s horse, *E. przewalskii*—warranting reevaluation of evidence for domestication. Here, we compare osteological traits hypothesized to have been caused by horse transport at Botai with wild Pleistocene equids in North America. Our results suggest that damage observed in Botai horse teeth is likely generated by natural disturbances in dental development and wear, rather than through contact with bridle equipment. In light of a careful reconsideration of the mid-Holocene archaeological record of northern Eurasia, we suggest that archaeological materials from Botai are most effectively explained through the regularized mass harvesting of wild Przewalski’s’ horses—meaning that the origins of horse domestication may lie elsewhere.

## Introduction

The innovation of horse transport—chariots and mounted horseback riding—transformed nearly every aspect of the ancient world. Horse carts and riding altered the trajectories of processes such as pastoral subsistence, mobility, warfare, communication, trade, agriculture, disease and biological exchange^1^. Despite the centrality of horse domestication to human history, however, a clear understanding of the initial domestication of *Equus caballus* has proven particularly elusive—in large part because of the vagaries of the archaeological record, which have left few material traces with which to understand early human-horse relations. As a result, archaeological models for horse domestication often relied largely on indirect measures such as the frequency of *Equus* bones at archaeological sites, or changes in their morphological variability, to distinguish human activity^2,3^.

Emerging techniques in archaeological science have helped to fill these analytical gaps, providing direct lines of evidence for human-horse interactions that can be identified in ancient material assemblages. Most significantly, the discovery of “bit wear”– damage caused by a bridle mouthpiece to the equine dentition^4,5^—helped crystallize the emerging discipline of equine paleopathology^6–9^. In the late 2000s, an archaeological consensus appeared to converge on sites of the Botai culture in northern Kazakhstan dating to the 4th millennium BCE, as the birthplace of horse domestication—based in no small part on the identification of apparent “bit wear” on a Botai tooth^10^.

In the last several years, though, continued innovation of new scientific methods –and their application to Botai materials has upended core assumptions of the Botai domestication model. Genomic sequencing demonstrated that Botai equids are in fact not the progenitor of *E. caballus* but a sister taxon, *E. przewalskii*^11^. This animal, the Przewalski’s horse, is today a wild species with threatened conservation status, and has never been managed or used for transport in the historic era. In light of continuing progress in our understanding of physiological and anatomical links between equine skeletal changes and human activity since the emergence of Botai consensus^12–14^, this discovery warrants a reconsideration of the argument for horse transport at Botai.

Here, through careful comparisons with the fossil record of Pleistocene wild horses in North America, we demonstrate that apparent instances of bit damage on Botai horses are likely generated by natural disturbances in dental development paired with natural wear. In light of new evidence and reassessment of other key tenets of the argument for horse management at Botai, we suggest that this important assemblage instead represents the final chapter of a millennia-long tradition of mass harvesting of wild horses. We offer an alternative model for the origins of the domestic horse that places *E. caballus* within a broader Eurasian and African trajectory of equid domestication and transport.

### Debates over horse domestication in the Trans-Urals

The earliest unambiguously managed specimens of the domestic horse, *E. caballus,* originate from the Sintashta culture in the Black Sea steppes and the Trans-Ural region of Russia, Kazakshtan, and Ukraine—where paired horse burials and partial remains of spoked wheel chariots can be found dating to the early decades of the 2nd millennium BCE^15^. While no specimens closely related to *E. caballus* have yet been identified that significantly predate this period^16^, many scholars have hypothesized that the first domestication of horses took place far earlier, during the Eneolithic or Copper Age. Specifically, influential works^17,18^ hypothesized that horse herding and riding both began as far back as the 4th millennium BCE—when archaeological and human genomic evidence attest to important population movements across Europe and Asia^19^. Around this time, many areas of northern Eurasia also experienced an apparent spike in the frequency of horse bones found at archaeological sites, which some interpret as evidence for human-mediated dispersal of domestic *E. caballus*^2,3^. At some sites, this pattern in skeletal frequencies is bolstered by the occasional recovery of horse-themed works of portable art, and identification of horse bones in ritual or funerary contexts^20^. None of these pre-Sintashta lines of evidence, however, uniquely distinguish a domestication relationship between humans and horses.

Early arguments for Eneolithic domestication in the Black Sea steppe or Eastern Europe were bolstered by the discovery of anthropogenic bit wear damage to the lower second premolar of a horse specimen from the site of Deriyevka in Ukraine, initially thought to date as early as ca. 4000 BCE^5^. However, direct radiocarbon dating of this horse revealed it to be an intrusion from the early Iron Age, ca. 700–200 BCE^21^, dashing any direct links between the site and human management of domestic horses, and dimming enthusiasm for Deriyevka as the epicenter for domestication.

**Figure 1:**
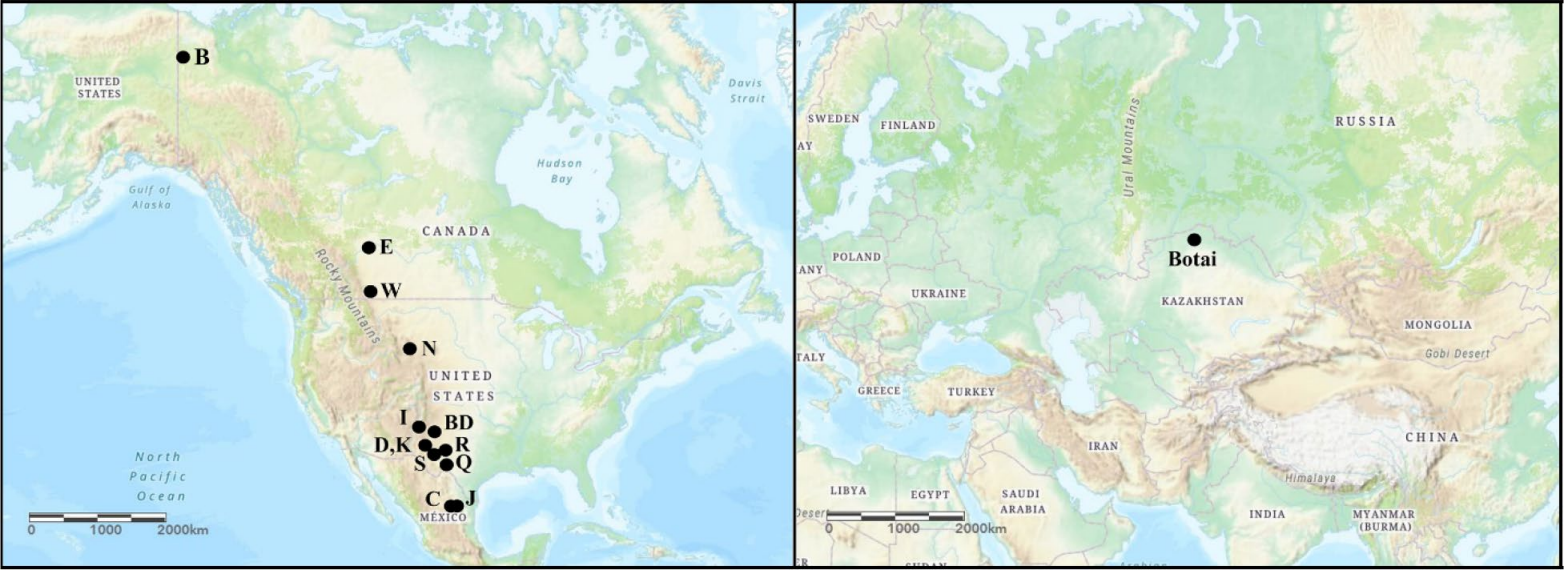
Geographic location of Botai (right) and North American Pleistocene sites (left). Site abbreviations: B = Bluefish Caves, BD = Blackwater Draw Loc. 1, C = Cedral, D = Dry Cave, E = Edmonton area gravel pits, I = Isleta Cave No. 2, J = San Josecito Cave, K = Dark Canyon Cave, N = Natural Trap Cave, Q = Cueva Quebrada, R = Scharbauer Ranch, S = Salt Creek, W = Wally’s Beach. This map was generated using the open- source software qGIS (https://www.qgis.org/en/site/), version 3.18.

In the early 2000s, increased scholarly attention coalesced around another archaeological site (and related localities of the same general period) as a potential candidate for Eneolithic horse domestication: Botai. This site is located along a tributary of the Ishim River along Kazakhstan’s northwestern border with Russia (Fig. 1). Here, researchers uncovered a pithouse village along with a massive assemblage composed nearly entirely of horse remains. Subsequent analysis of the site, and related assemblages from the sites of Krasni-Yar and Vasilovka, revealed intricately decorated horse bones, tools made from horse bones^22^, and pithouses filled with decayed vegetative matter hypothesized to be horse dung^23^. At some Botai locations, posthole structures raised the possibility of corrals^22^. Analysis of some loose lower premolars from Botai even suggested damage caused by a bit, although not in the typical form expected from a metal mouthpiece^24^. Conveniently positioned both chronologically and geographically, Botai seemed to slot nicely into extant explanatory models for Indo-European movements and help explain the broader cultural transitions observed in Eurasia during the 4th millennium BCE.

Nonetheless, initial response to the Botai domestication hypothesis was far from one of consensus. As archaeologists developed an ever-expanding toolkit for identifying evidence of human activity in the archaeozoological record, a number of concerns emerged which cast serious doubt on the site’s viability as an early domestic horse assemblage. One of the most serious among these concerns was the apparent inconsistency of age and sex patterns in the Botai horse assemblage with those of a pastorally-managed herd. As articulated by Levine, through her detailed ethnographic study of contemporary pastoral groups^8^, pastoral management of a breeding herd typically entails slaughter of two categories of animal: (1) adults that have reached the age of reproductive senescence, these being mostly female, and (2) juvenile animals that have yet to reach breeding age, these being mostly male. The resulting bimodal mortality profile has proven to be a consistent feature of pastoral horse assemblages from the Bronze Age onwards^25,26^. In contrast, demographic analysis of Botai horses by Levine^8^ and a reanalysis by Olsen^22^ revealed that the Botai horses are primarily breeding-age adults, split into a roughly equal balance of male and female horses. Most recently, this finding was confirmed a third time through DNA study by Fages et al.^27^, who found a marked contrast between the sex ratios found in Botai and later ritual assemblages of domestic horses. Although it is possible that the inclusion of a large number of ritually-sacrificed, adult male transport horses into an otherwise predominantly female assemblage could have artificially skewed the age and sex patterns^22^, at face value, the overall mortality patterns found at Botai appear fundamentally inconsistent with pastoral management.

Other lines of evidence also seemed to cast a shadow of doubt on the domestic status of Botai horses. As a more nuanced understanding of the process of equine tooth wear emerged, Olsen^22^ demonstrated that irregularities in occlusal morphology identified on teeth from Botai were not necessarily indicative of bit use, as similar features can be identified on wild equids. And while vertebrae from early riding horses often preserve regular traces of damage to the lower back^9^, pathological study of the small number of available vertebral elements from Botai^28^ showed no recognizable damage associated with horse transport. Finally, bone arrowheads were actually found in direct association with at least some specimens—demonstrating that the animals were killed through hunting^22^. Benecke and von den Driesch^29^ found that in terms of size and morphological variance, Botai specimens were not separable from earlier hunted assemblages of wild horses—which have formed a key aspect of northern Eurasian subsistence since the Paleolithic. As a result of these considerations, even researchers that favored a Botai domestication model were compelled to admit that managed/ridden horses must have comprised a small percentage of the assemblage^20,22^.

### The emergence of a Botai consensus

Amidst these conflicting signals, two new discoveries in the 2000s tilted the needle in favor of popular consensus for domestication at Botai. The first of these was the publication of a new dataset of isotopic measurements from lipid residues in ceramic sherds by Outram et al.^10^. An earlier study using the same approach^30^ identified that the ceramic vessels at Botai were used primarily to process horse fats—a conclusion that was perhaps unsurprising, given the overwhelming emphasis on horses in the faunal assemblage. In the updated study, Outram and colleagues sought to differentiate fats linked with milk products from those linked to meat and adipose tissue. New reference datasets showed that while horse milk and meat fats had an overlapping distribution in carbon isotope values, the seasonally-sensitive hydrogen isotope deuterium could help identify residues linked to the summer months, when milking typically occurs. Together, these observations formed the basis of an argument that Botai ceramics once held milk, rather than other horse fats.

A second crucial component to this study was a reanalysis of jaw bones and dentition, using revised criteria developed by Bendrey^6^ that sought osteological features more reliably associated with horse transport than those identified by Brown and Anthony^24^ but criticized by Olsen^22^. These new criteria, derived through careful comparison of natural history collections, included new bone formation to the diastema of the lower jaw, a feature thought to occur in very low levels in wild animals, and the erosion of cementum and enamel along the anterior margin of the lower second premolar. While such cementum erosion occurs in both wild and domestic animals, by comparing wild and domestic animals with known life and work histories, Bendrey^6^ found that metal bits often produce a version of this damage that is parallel-sided in shape, and is often more invasive than natural wear (causing exposure of dentine).

**Figure 2:**
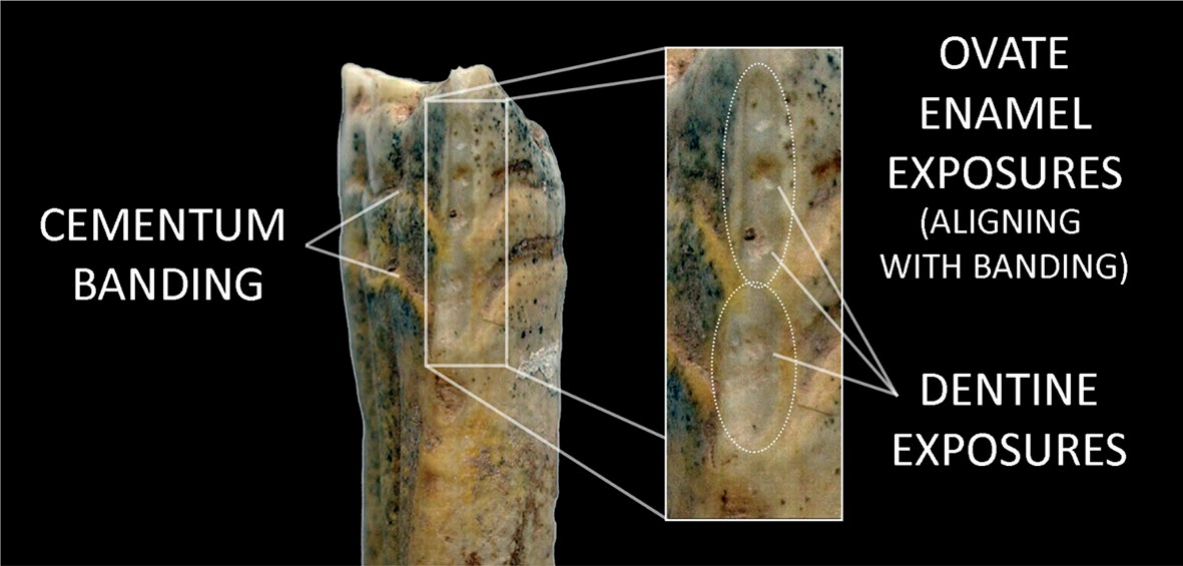
Botai horse tooth cited as conclusive evidence of bit wear in Outram et al.^10^, showing the existence of two overlapping areas of enamel exposure corresponding to areas of reduced cementum deposition along the buccal and lingual margins.

Application of these new criteria to Botai revealed some instances of new bone formation on the lower jaw that were higher than those observed in wild comparative collections (a score of “2” in the categorical scoring system). Additionally, one lower second premolar seemed to demonstrate long, parallel-sided cementum erosion and enamel wear resulting in dentine exposure—a new “smoking gun” for horse bridling and riding during the 4th millennium BCE (Fig. 2). These new lipid values and paleopathological finds were further supported by metric comparisons of metapodial bones, which appeared to show that the Botai horses were the domestic *E. caballus*^10^. Together, these new finds underscored an emerging consensus that Botai represented the first step in the domestication story, which persists to the present day.

### Recent innovations in archaeological science

Although the consensus forged by these discoveries was robust, recent discoveries necessitate a reevaluation of some or all of the Botai domestication argument. While morphological distinction among “caballine” horses (*Equus caballus* and its close relatives) is notoriously challenging, improving techniques in ancient DNA now enable full genomic sequencing of ancient specimens—permitting for the first time a reliable species identification of archaeological equids. In a key finding, Gaunitz and colleagues^11^ revealed that despite the previously identified morphological patterns in metapodial shape, all analyzed Botai specimens were in fact *E. przewalskii*, and not the progenitor of the domestic horse. Attempting to reconcile this discovery with the previous consensus for domestication, Gaunitz and coauthors concluded that Botai must represent an earlier domestication event that was later abandoned, leading to the subsequent feralization and rewilding of the Przewalski’s horse.

The contextual nature of most of the existing evidence for domestication at Botai, however, may not support this interpretation. Many assumptions underpinning this earlier work—such as the idea that vegetal matter found in pithouses was horse dung, that postholes were used to form corrals, or that summer seasonality of isotope values indicated horse milk—were contingent upon the presence of domestic horses, rather than empirically demonstrated. Other recent biomolecular work, such as a recent study analyzing dental calculus of human remains from Botai^31^, found no evidence for dietary milk proteins of any kind. Consequently, it seems that the argument for Botai domestication of *E. przewalskii* now hinges heavily, if not solely, on the mandibular bone formation and apparent bitting damage found on a single lower second premolar.

### Re-examining evidence for horse transport at Botai

How robust is the evidence for horse transport at Botai? A careful examination of published imagery of this tooth shows evidence of natural damage and disturbances in dental development that may undercut the argument linking the apparently parallel-sided anterior enamel and dentine exposure observed in a single second premolar from Botai with a bridle bit. Specifically, although the enamel exposure visible along the anterior margin of this tooth does indeed appear parallel-sided at first glance, it actually consists of two adjoining, oval-shaped areas of exposure. These exposed areas of enamel correspond precisely with bands or “valleys” of reduced cementum deposition visible along the buccal and lingual margins of the tooth (Fig. 2). Reduced cementum deposition at the occlusal/coronal area of the tooth presumably would make the anterior side of the premolar more susceptible to cementum erosion from natural wear processes, resulting in the exposure of two oval-shaped areas of enamel. The exposed area of enamel is also interrupted by a series of small circular pits in the enamel, producing small areas of exposed dentine, which Outram et al.^10^ argue to be indicators of bit use—a marker of severe and invasive wear that is not observed in unworked horses^6^. However, this photo shows that each of the pits in the Botai tooth margin are exceedingly small in diameter—far smaller than any we have observed in archaeological horse remains from Mongolia, even those controlled with organic bits^32^. Small, circular pitting is also a characteristic sign of pit-form enamel hypoplasia—a dental defect caused by disruption of normal enamel matrix secretion, which results in pockets of thin or poorly developed enamel. These observations raise the possibility that, in a large assemblage of wild horses, natural processes (rather than human activity) could produce similar features to those identified in the Botai assemblage.

## Methods

To characterize the frequency of damage found on the Botai tooth (cementum banding, as well as the frequency of enamel hypoplasia, and pitting of the enamel along the anterior margin of the second premolar) in wild populations, we analyzed a sample of 72 lower second premolars and 81 upper second premolars of wild equids (*Equus* spp.) derived from Pleistocene fossil localities in North America—where no domestic horses existed prior to the arrival of European colonists in the last millennium. Our analyzed sample included material from Bluefish Caves in northern Yukon, various localities in the Edmonton area and the Wally’s Beach site in Alberta, Natural Trap Cave from the Bighorn Mountains of northern Wyoming, various localities in New Mexico and Texas, and Cedral and San Josecito Cave in northeastern Mexico (Fig. 1). All of these sites preserve abundant equid remains, which are late Pleistocene in age^33^. The specimens we studied are housed in the collections of the Canadian Museum of History (CMH; Gatineau, Quebec, Canada), Instituto Nacional de Antropología e Historia (INAH DP; Mexico City, Mexico), Los Angeles County Museum of Natural History (LACM; Los Angeles, California, U.S.A.), Royal Alberta Museum (RAM; Edmonton, Alberta, Canada), University of Kansas (KU; Lawrence, Kansas, U.S.A), and University of Texas-Austin (Vertebrate Paleontology Laboratory [TMM] and Texas Archaeological Research Laboratory [AMIS]; Austin, Texas, U.S.A.) and El Paso (UTEP; El Paso, Texas, U.S.A.). All of the specimens were examined with a hand lens using oblique lighting. Our study sample included worn and unworn teeth.

### Enamel hypoplasia and cementum banding

For those specimens showing hypoplastic defects, we noted the location of the defect on the tooth crown. We identified the type of defect(s) present on the anterior margin of the tooth (i.e., pit-form, plane-form, or furrow-form; following Witzel et al.^34^ and Hillson^35^) and whether they presented dentine exposure. We treated isolated teeth as separate individuals, unless there was evidence that isolated teeth were associated (i.e., left and right teeth of the same mandible or palate), in which case the associated teeth were treated as a single individual. We calculated the percentage of specimens (i.e., individuals) presenting enamel hypoplasia with and without dentine exposure on the anterior margin of the lower and upper second premolars (Table 1; Supplementary Table 1).

**Table 1:** Frequency of enamel hypoplasia on anterior side of lower and upper second premolars in study samples from Pleistocene sites in North America.

Tooth	Localities	# Studied	# With anterior hypoplasia of any type [frequency (%)]	# With pit-form anterior hypoplasia and dentine exposure [frequency (%)]
Lower P2	Bluefish Caves	12	2 (16.7)	2 (16.7)
	Natural Trap Cave	17	6 (35.3)	4 (23.5)
	Edmonton area and Wally's Beach	5	1 (20.0)	1 (20.0)
	Various localities in New Mexico and Texas	13	2 (15.4)	2 (15.4)
	Cedral and San Josecito Cave	25	3 (12.0)	2 (8.0)
	All localities combined	72	14 (19.4)	**11 (15.3)**
Upper P2	Bluefish Caves	12	1 (8.3)	0 (0.0)
	Natural Trap Cave	39	3 (7.7)	2 (5.1)
	Edmonton area and Wally's Beach	2	0 (0.0)	0 (0.0)
	Various localities in New Mexico and Texas	9	0 (0.0)	0 (0.0)
	Cedral and San Josecito Cave	19	0 (0.0)	0 (0.0)
	All localities combined	81	4 (4.9)	**2 (2.5)**

Bold value indicates the frequency of specimens with anterior pit-form hypoplasia with dentine exposures

We note that one obstacle to the study of enamel hypoplasia in the cheek teeth of equids and other hypsodont ungulates is the presence of cementum covering the tooth crown^36,37^. In living horses, the cementum is thicker at the occlusal/coronal area (i.e., the extra-alveolar area) of the tooth, particularly on the buccal side of the lower cheek teeth and the lingual side of the upper cheek teeth^38,39^. Cementum in the occlusal/coronal area of the tooth is primarily comprised of tertiary cementum and is attached to the gingival mucosa^38^. Cementum significantly decreases in thickness below the occlusal/coronal area (i.e., at the intra-alveolar area of the tooth) in a cervical direction where, depending on the ontogenetic development of the tooth, it might be absent^38,39^. Aged horses with teeth in extreme stages of wear often show abnormally large increases in tertiary cementum^38^.

To control for the impact of variable cementum coverage, we qualitatively scored the extent to which cementum covers the tooth crown using a 0–5 scoring system (following Barrón-Ortiz et al.^40^) (Supplementary Table 1). In this system, 0 indicates that the tooth crown is not covered by cementum, 1 denotes that 1–25% of the tooth crown is covered by cementum, 2 indicates that 26–50% of the tooth crown is covered by cementum, 3 denotes that 51–75% of the tooth crown is covered by cementum, 4 indicates that 76–95% of the tooth crown is covered by cementum, but that the cementum present below the occlusal region of the tooth consists of a thin layer, and 5 denotes that the entire tooth crown is covered by a thick layer of cementum. We excluded any specimens with a score of 5 from our analysis, because the cementum covering the tooth made it difficult to consistently evaluate whether enamel hypoplasia was present in the tooth. For teeth with only one side of the tooth crown completely covered by cementum we scored the exposed sides of the tooth. Cementum did not pose a serious problem to the examination of enamel hypoplasia for many of the teeth we studied, because post-mortem weathering and degradation of cementum exposed the enamel underneath.

For cementum banding, we evaluated the presence/absence and number of cementum bands (when present) at the occlusal/coronal area of the tooth (Supplementary Table 1). We treated isolated teeth as separate individuals and associated teeth were treated as a single individual. We excluded teeth in which the occlusal/coronal cementum was damaged or weathered from cementum analysis, resulting in a total sample of 52 lower and 78 upper second premolars. From this sample, we calculated the proportion of specimens displaying two or more well-defined cementum bands (Table 2).

**Table 2:** Frequency of multiple, well-defined cementum bands in the occlusal/coronal area of upper and lower second premolars in study sample of North American Pleistocene equids.

Sample	# Studied	# With 2 or more cementum bands	Frequency (%) of teeth with 2 or more cementum bands
Upper P2	78	20	25.6
Lower p2	52	8	15.4

### Mandible bone formation

Although preservation from Pleistocene horses is generally too poor to assess diastema damage within this same assemblage, we studied six horse mandibles from the well-preserved Wally’s Beach site in southern Alberta (Table 3; specimens housed at the RAM) to determine whether they present diastema bone formation comparable to levels observed in the Botai assemblage. We used the scoring system proposed by Bendrey^6^ to determine the extent of new bone formation in the diastema.

**Table 3:** Diastema bone formation score in six Pleistocene horse mandibles from the Wally’s Beach site, Alberta.

Specimen number	Element	Diastema bone formation score
RAM DhPg-8: 861.1 (Horse 3)	Left and right mandibles	2/1
RAM DhPg-8: 863 (Horse A)	Right mandible	1
RAM DhPg-8: 864 (Horse A)	Left mandible	1
RAM DhPg-8: 876.1 (Horse B)	Left and right mandibles	–/–
RAM DhPg-8: 69.1 (Horse D)	Left and right mandibles	1/1
RAM DhPg-8: 74.1 (Horse C)	Left mandible	–
RAM DhPg-8: 3437.2 (Horse 2)	Left and right mandibles	0/1

## Results

**Figure 3:**
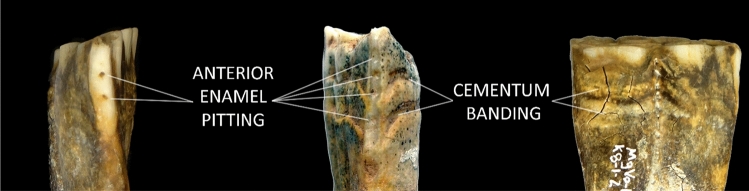
Comparison of the Botai “bit wear” specimen (center) with a similar tooth from a Pleistocene wild horse from North America (specimen n^o^ MgVo-1: K8-1-2; Canadian Museum of History) that exhibits anterior enamel pits with dentine exposure (left) and cementum banding on both the buccal surface (right) and the lingual surface (not pictured).

Our results provide clear indication that pitting similar to that observed on the Botai horse is particularly common on the anterior margin of the lower second premolar of wild North American horses. Although only a small handful (n = 4; 2.5%) of specimens exhibit any form of visible enamel hypoplasia on the anterior margin of the *upper* premolar, on the *lower* premolar, a total of 14 specimens out of 72 (19.4%) teeth analyzed exhibit enamel hypoplasia (Table 1). Of these, 11 (15.3%; Table 1) lower second premolars display pit-form hypoplastic defects accompanied by circular or oval-shaped dentine exposures; a condition that is consistent with the damage observed in the Botai horse tooth (Fig. 3).

Furthermore, our evaluation of cementum banding, indicates that among wild horses, there is often a wide gradient in this trait—ranging from teeth that do not show any distinct cementum bands to those that show well-defined cementum bands. Teeth with at least two or more well-defined cementum bands, as is the case in the Botai tooth, appear to be slightly more common in the upper second premolar (25.6%) than in the lower second premolar (15.4%) (Table 2).

We were able to assess four out of six horse mandibles from the Wally’s Beach site for evidence of mandibular bone formation. Three of the four mandibles showed bone changes in the diastema indicative of a score of “1” in the scoring system developed by Bendrey^6^. These mandibles show faint changes, including the development of a well-defined ridge along the diastema and roughening of the bone. The remaining specimen that was well-preserved enough for us to study, showed well-developed pathological bone formation on the left side of the mandible, earning a score of “2” (Table 3). At Botai, 4/15 mandibles showed a similar score^10^.

## Discussion

Comparing our results to the Botai tooth provides strong indication that the osteological features taken for evidence of horse transport, may have been produced solely through natural processes. Although our analysis suggests that it is uncommon for enamel hypoplasia pits to co-occur in multiples on the anterior margin of the tooth, one wild horse in our sample, from Bluefish Cave I (MgVo-1) in northern Yukon, exhibited two such pits, nearly identical to those attributed to bit wear at Botai, along with visible cementum banding (Fig. 3). Based on available data, we are unable to speculate on whether enamel hypoplasia itself might occur in higher frequency or severity in domestic and wild samples. However, the high frequency of enamel hypoplastic defects with dentine exposed in North American horses suggests that dentine exposure—in the form of circular or oval pits—cannot be considered reliable evidence of transport damage. In fact, the apparent frequency of this type of damage in the Botai assemblage (1/9 or 11.1%^10^) is commensurate or even below the frequency of this type of feature observed in wild North American populations (15.3%; Table 1).

**Figure 4:**
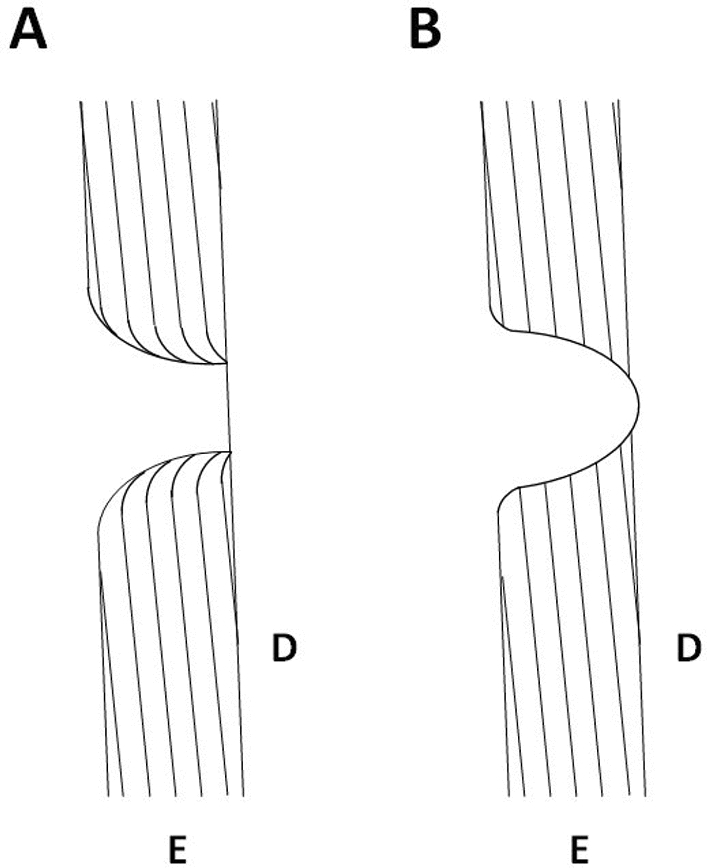
Schematic diagram of two longitudinal histological sections showing the differences between (**A**) a pit-form hypoplastic defect and (**B**) a pit produced by other processes such as flaking of poorly mineralized enamel or enamel erosion due to mechanical wear. (**A**) In a pit-form hypoplastic defect the rim of the pit is smoothly curved and the striae of Retzius in the enamel forming the walls of the pit are also curved. (**B**) A pit produced by flaking of poorly mineralized enamel or enamel erosion due to mechanical wear cross-cuts normal striae of Retzius and it usually lacks a smooth and curved rim. E = enamel; D = dentine; lines in enamel represent striae of Retzius.

Because of the different processes involved in bit wear and hypoplastic defects, the Botai tooth can potentially be evaluated in a non-destructive manner using recently developed visualization and virtual histological techniques^40^. In contrast to other types of enamel hypoplasia, pit-form hypoplastic defects are characterized by a scatter of isolated pits or single pits surrounded by unaffected enamel^34,35^. The rim of each pit, as seen on the surface of the tooth, is usually smooth^35^. In longitudinal histological sections, the rim of each pit appears smoothly curved and the striae of Retzius in the enamel forming the walls of the pit are also curved^34,35,41^. Frequently accentuated lines, i.e., Wilson bands, are also seen associated with pit-form hypoplastic defects^34,35^. Pits that are caused by other factors such as flaking of poorly mineralized enamel^42–44^, cavitary lesions resulting from pre-eruptive enamel resorption^41^ and enamel erosion from mechanical wear or abrasion (e.g. bitting damage) lack a smooth and curved rim and they cross-cut normal striae of Retzius (Fig. 4). Consequently, damage caused by bits should be easily distinguishable from pit-form hypoplastic defects using histological approaches.

Interestingly, our results suggest a discrepancy in the frequency of enamel hypoplasia in the anterior margin of the upper and lower second premolars of the wild equid samples (Table 1). These teeth form at approximately similar times during ontogeny^45^. Therefore, disruptions in enamel secretion would be (in theory) expected to be correlated to some degree in upper and lower second premolars for a given individual. Although we were not able to analyze associated upper and lower second premolars in our sample, there is no indication of preservation bias, collecting bias, or sampling biases between upper and lower teeth in the samples we studied. One possible explanation for this discrepancy in the frequency of enamel hypoplasia in the upper and lower teeth is different dental developmental geometries. Dental developmental geometry is a factor that is known to affect the expression and visibility of enamel hypoplasia^35^.

Our new data suggest that, without careful consideration of other enamel and cementum patterns, criteria used to identify bit wear through enamel exposure can sometimes produce spurious results. Standard practice suggests that cementum erosion on the anterior margin of the occlusal/coronal area of the lower second premolar, leading to the exposure of a band of enamel that is parallel-sided in shape and that has a height greater than 5 mm (measured from the occlusal surface towards the mandible), is indicative of bit damage^6^. The Botai tooth presents a band of enamel exposure that is 18.0 mm in height^10^, well above the cutoff value of 5 mm. However, as indicated previously, despite appearances of parallel sides, the enamel exposure consists of two adjoining, oval-shaped areas of exposure. These exposed areas of enamel correspond with two well-defined bands of reduced cementum deposition, observed along the buccal and lingual sides of the tooth. We propose that reduced cementum deposition in this region of the tooth made the anterior side of the premolar more susceptible to cementum erosion from natural wear, resulting in the exposure of two tall, oval-shaped areas of enamel.

Multiple, well-defined cementum bands are relatively common in the lower (15.4%) and upper (25.6%) second premolars of wild equid teeth we studied (Table 2). In some cases, cementum bands appear to be the result of reduced cementum deposition, similar to the condition observed in the Botai tooth. However, in other cases they appear to result from increased cementum deposition on top and below the cementum band. Based on these observations, two alternative, although not mutually exclusive, mechanisms can potentially explain the formation of cementum bands. The first of these is disruption of the secretory activity of cementoblasts, the cells that form cementum—if tooth eruption is more or less constant, disruption in the secretory activity of cementoblasts would lead to a reduction in the deposition of tertiary cementum for the duration of the disruption. Alternatively, cementum bands may be caused by changes in the rate of tooth eruption—if the deposition of tertiary cementum is more or less constant, then changes in the rate of tooth eruption would affect how much tertiary cementum gets deposited around the occlusal/coronal area of the tooth crown at a given time (less cementum if the rate of tooth eruption is increased, and more cementum if the rate of tooth eruption is decreased). In either case, it would seem that the mechanism of cementum band formation is common enough in wild horses to warrant careful consideration by archaeologists interested in anthropogenic tooth damage.

**Figure 5:**
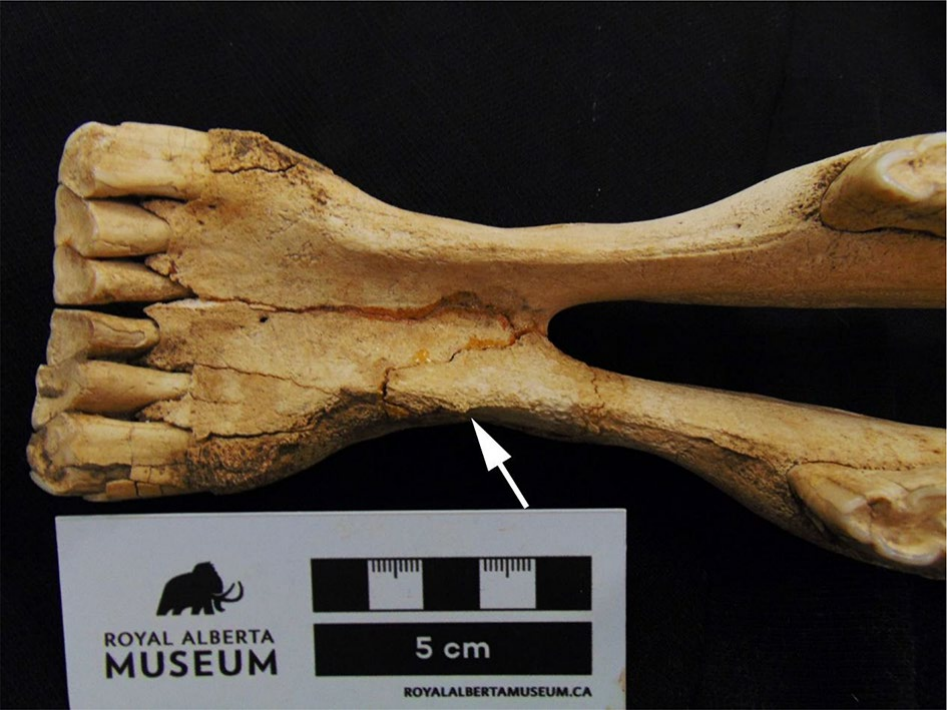
Mandibular bone formation on wild Pleistocene horse (specimen n^o^ DhPg-8: 861.1; Royal Alberta Museum) from Wally’s Beach, Alberta, earning a score of “2” on the system devised by Bendrey^6^.

Although we were unable to confidently assess the relative frequency of mandibular bone formation on the diastema in our sample due to the poor preservation of this element in fossil contexts, one of four analyzable specimens from our best-preserved assemblage (Wally’s Beach, Alberta) exhibited well-developed pathological bone formation—earning a score of “2” using the scoring system outlined by Bendrey^6^ (Table 3; Fig. 5). Although the precise cause for this bone formation in the Wally’s Beach horse is not immediately clear, diet and age likely played a factor—based on dental wear, this horse is elderly (likely 15 + years based on the triangular shape of the incisor grinding surface and the absence of cups/interior enamel spots in the central incisors). The other three Wally’s Beach specimens well-preserved enough to be analyzed exhibited a score of “1”, while two more were too damaged to be confidently assessed (Table 3). The diastema score from Wally’s Beach is equal to those previously taken to represent evidence of bit damage at Botai^10^, and occurs in a roughly comparable frequency (1/4) to the Botai assemblage (4/15).

### Alternative explanations for Botai

In light of our new data, arguments for horse domestication at Botai no longer appear to be supported by the available archaeological evidence. Without the presumption of horse transport, many aspects of the Botai assemblage are more efficiently explained by interpretation of the site as the result of regularized mass-harvesting of wild horses. For example, Botai’s location at a river crossing is consistent with wild equid hunting tactics that date back deep into the Pleistocene. At Paleolithic sites across Europe, entire bands of horses—either mostly-female harem groups, all-male bachelor bands, or both—were commonly ambushed alongside natural water features where they were more effectively trapped and slaughtered^46^. This strategy appears to have been employed by the earliest hominin horse hunters, dating back nearly a half million years or more^47,48^. Group harvesting at Botai could easily explain unresolved questions in the assemblage, including apparent presence of entire carcasses, the predominance of prime-aged adult animals, and the recovery of bone arrowheads in situ with deceased horse remains, as well as the utter absence of other domestic fauna at Botai^22^. The relatively equal ratios of male and female animals found at Botai could imply that the site was used to harvest both bachelor bands and harem groups over its use history. Summer seasonality identified by Outram et al.^10^ using isotope data could reflect horse milk production, but many mass harvesting sites of horses also display consistent summer seasonality, even over centuries or millennia of reuse^49^. If chosen for a favorable topographic position or location on a key ecological corridor or migration route, horse mass harvesting sites may have been regularly utilized in a particular time of year over long stretches of time.

A reappraisal of the pre-Botai archaeological record of humans and horses also supports this view. Many of the cultural modifications found in the Botai artifact assemblage—the decoration of horse bones, the use of horse bones as tools, and even the occasional ritual inhumation of horse remains—are fully consistent with hunter-gatherer cultures in which horse hunting plays an important role. Horses are the most commonly depicted animal in Eurasian Paleolithic cave paintings^50^, and were a favorite muse for hunter-gatherer artists across the Pleistocene and into the Holocene—appearing on bones, ivory, or stone objects—and probably many organic artifacts that have not survived—as decorations or as dedicated votives^51^. The discoveries of ritual features and artwork at Botai or Eneolithic sites from the Black Sea region, while important, fail to effectively delineate a domestication relationship from the rich hunting tradition that preceded it.

How, then, to explain the apparent resurgence in the frequency of horses—a Pleistocene animal whose populations began to dwindle after the Last Glacial Maximum—during the 4th millennium BCE? A convergence of climate and human factors unrelated to horse domestication may have contributed. The late Neolithic witnessed a cooling trend across much of northern Eurasia^52–54^ that could have boosted the population size and expanded the viable habitat of cold-adapted equids. These favorable climatic changes may also have been buoyed by the behavior of pastoralists migrating into new areas of Europe and Asia, who likely slashed forests and increased the cover of grassland habitats across a wide region^55^.

Removing Botai from archaeological narratives streamlines and improves other emerging models for Eurasian prehistory. For example, if Botai people were horse hunters and horses were not yet domesticated ca. 3500 BCE, the absence of human genomic links between Botai and pastoralist Yamnaya people^56^, and the absence of domestic horses south of the Caucasus prior to 2000 BCE^57^ are consistent with predictions, rather than lingering puzzles.

Finally, it is important to note that the interpretation of Botai is not simply academic, but has profound practical implications for conservation. *Equus przewalskii* is now a badly endangered taxon that only a few decades ago was considered extinct in the wild. Thanks in part to careful conservation efforts in Mongolia and elsewhere, this animal has been painstakingly reintroduced from a host population of less than a dozen individuals^58^. Since the conclusion of Gaunitz et al.^11^ that Przewalski’s horses are not truly “wild” is not supported by the available archaeological data, their protection must remain an essential priority for conservation of grassland biodiversity in the Eurasian steppe.

### Alternative models for horse domestication

If Botai does not represent either *E. caballus* or the origins of horse transport, where, then, are we left to turn for answers to the initial domestication of the horse? Future work will be necessary to answer this question with any confidence. However, we suggest that more careful attention should be paid to the domestic horses and chariots of Sintashta, and the preceding cultures of the Black Sea steppe, where horse milk proteins have recently been directly identified in human teeth dated to the 3rd millennium BCE^31^. We also point to the likely significance of cultural connections between Transcaucasia and adjoining areas of the Near East, where donkeys and onagers were used for transport by the 3rd millennium BCE or before^59,60^. In settling on a new model for horse domestication, it will be essential to revisit the findings of a rapidly changing discipline of archaeological science with fresh eyes and a willingness to reevaluate old conclusions based on new discoveries.

## Conclusion

The continual emergence of new lines of evidence for understanding ancient human-horse interactions necessitates vigilant reevaluation of models for the domestication of the horse. Based on comparison of materials from the site of Botai with wild equid remains from North America, we suggest that skeletal changes to the diastema and lower premolars observed in this assemblage are a result of natural processes rather than early transport. In light of these finds and other recent biomolecular discoveries, this site and its related assemblages appear to be best explained through mass harvesting of wild horses rather than early domestication. Future work will continue to require careful reanalysis of existing assumptions and revisitation of inherently ambiguous and indirect archaeological data.

## Supplementary Information

Supplementary Information

## Data Availability

All data used in the analysis are provided directly in the manuscript and its supporting information.
